# Expression patterns of eight RNA-modified regulators correlating with immune infiltrates during the progression of osteoarthritis

**DOI:** 10.3389/fimmu.2023.1019445

**Published:** 2023-03-15

**Authors:** Ziyi Chen, Wenjuan Wang, Yinghui Hua

**Affiliations:** Department of Sports Medicine, Huashan Hospital, Fudan University, Shanghai, China

**Keywords:** osteoarthritis (OA), RNA modification regulators, immune infiltration, m7G methylation, alternative polyadenylation (APA), m6A methylation

## Abstract

**Background:**

RNA modifications in eukaryotic cells have emerged as an exciting but under-explored area in recent years and are considered to be associated with many human diseases. While several studies have been published relating to m6A in osteoarthritis (OA), we only have limited knowledge of other kinds of RNA modifications. Our study investigated eight RNA modifiers’ specific roles in OA including A-to-I, APA, m5C, m6A, m7G, mcm5s2U, Nm and Ψ together with their relationship with immune infiltration.

**Methods:**

RNA modification patterns in OA samples were identified based on eight-type RNA modifiers and their correlation with the degree of immune infiltration was also methodically investigated. Receiver operating characteristic curves (ROC) and qRT-PCR was performed to confirm the abnormal expression of hub genes. The RNA modification score (Rmscore) was generated by the applications of principal component analysis (PCA) algorithm in order to quantify RNA modification modes in individual OA patients.

**Results:**

We identified 21 differentially-expressed RNA modification related genes between OA and healthy samples. For example, *CFI, CBLL1* and *ALKBH8* were expressed at high levels in OA (P<0.001), while *RPUSD4, PUS1, NUDT21, FBL* and *WDR4* were expressed at low levels (P<0.001). Two candidate RNA modification regulators (*WDR4* and *CFI*) were screened out utilizing a random forest machine learning model. We then identified two distinctive RNA modification modes in OA which were found to display distinctive biological features. High Rmscore, characterized by increased immune cell infiltration, indicated an inflamed phenotype.

**Conclusions:**

Our study was the first to systematically reveal the crosstalk and dysregulations eight-type of RNA modifications in OA. Assessing individuals’ RNA modification patterns will be conductive to enhance our understanding of the properties of immune infiltration, provide novel diagnostic and prognostic biomarkers, and guide more effective immunotherapy strategies in the future.

## Introduction

Osteoarthritis (OA) is the most common arthritis leading to ache, joint destruction, and eventually handicap; women and the aged are disproportionately affected by this disease (1–3). The combined effects of an aging global population, the increasing incidence of obesity and numbers of joint trauma have led to this disease becoming much more prevalent, with a minimum of 500 million people estimated to be affected by OA worldwide (1, 2). This disease imposes a substantial health burden on the individuals, health-care systems and socioeconomic system (4). Clinical diagnosis involving a combination of basic symptoms and a brief physical examination is the gold standard for confirming OA (1); sometimes, a plain radiograph is needed for diagnosis if the clinical manifestation is atypical (5). However, effective diagnostic criteria at early stage are missing. Nowadays typical treatment is characterized as palliative and reactive, rather than proactive and preventive (1). Joint replacement surgery can be applied when OA advances into end-stage but is accompanied by high mortality, complications and the risk of symptom reservation (6–9). Altogether considering the increased number of individuals affected by OA, the lack of an option to diagnose OA early, the absence of effective treatments, there is a clear and urgent need to figure out novel biomarkers in diagnosing OA.

Despite the lack of early diagnosis and a limited understanding of the OA pathogenic mechanisms, a growing body of clinical and experimental evidence has shown how crucial immune cells and immunological-related pathways are to the development of OA (10). Studies demonstrated that excessive inflammatory response participates in the progression of OA with innate immune cells taking part in the early inflammatory response and adaptive immune cells contributing to the chronic and relapsing course of inflammation (11–13). Therefore, analyzing the important roles played by immune cells and immunological-related pathways in the development of OA may offer a potential avenue for OA patients’ diagnosis and care.

As is well-known, the epigenetic modification plays a significant role in directing and maintaining distinctive cellular phenotypes (14). With advances in multiple sequencing technology, the role of RNA modifications in the happening and progression of many diseases such as multiple cancers, cardiovascular disorders, metabolic diseases, mitochondrial-related defects and so on has been increasingly elucidated (15). Particularly, it has been suggested that inflammatory diseases including OA may be related to RNA modification (16). Up to now, more than 170 types of RNA modifications have been detected in eukaryotes; many of these have been shown to be strongly related to various diseases (17). N6-methyladenosine (m6A), N1-methyladenosine (m1A), 5-methylcytidine (5mC), 7-methylguanosine (m7G), alternative polyadenylation (APA), 2′-O- methylation (Nm), uridine-to-pseudouridine (Ψ), adenosine-to-inosine transition (A-to-I), and 5-methoxycarbonylmethyl-2-thiouridine (mcm5s2U) have all been commonly investigated over recent years for their respective roles in the progression of disease (14, 18–20). Previous research found that targeted inhibition of m6A regulator—METTL3 could attenuate the senescence of synovial fibroblasts and limit OA progression (21). However, there is a lack of similar studies relating to other types of RNA modifications.

Molecular classifications of OA could accelerate the development of pre-diagnosis and individual-based target therapeutics for patients. For example, Yuan et al. grouped OA patients into four subpopulations with different biological and clinical features (glycosaminoglycan metabolic disorder, collagen metabolic disorder, activated sensory neuron and inflammation) according to the unsupervised clustering analysis of the cartilage transcriptome (22). Lv et al. also proposed the novel knee OA (KOA) molecular classification that was able to make a diagnosis of early KOA patients, predict high-risk KOA individuals, select individual-based appropriate therapy and assess therapeutic efficacy (23). However, as yet, nothing is known about the molecular modification of OA based on RNA modification.

Therefore, we are the first to identify RNA modification patterns in OA patients, comprehensively explore their correlation with immune infiltration and establish a specific rating system to quantize individuals’ pattern. Our study emphasized the significance of RNA modification in OA and offered potential therapy for OA patients in the future.

## Materials and methods

### Data source and differentially expressed genes acquirement

We downloaded the GSE51588 dataset containing 30 OA and 10 normal sub-chondral samples from the Gene Expression Omnibus database (GEO, https://www.ncbi.nlm.nih.gov/geo/). Our study selected nine types of RNA regulators that have been widely analyzed (14, 18, 19). The nine gene sets of 109 RNA modification regulators were previously identified by Mao et al. (14) and Chen et al. (19), and are listed in **Table S1**. Finally, a gross of 44 RNA regulators, belonging to eight types of RNA modifications (m6A, APA, m5C, Nm, m7G, Ψ, A-to-I and mcm5s2U) were included in our study. These modifiers consisted of 4 erasers, 8 readers and 32 writers.

Differentially-expressed RNA modification regulators between OA and normal were screened out by the application of the “limma” package of R software (version 4.1.1) with the criteria setting as a |log fold change (FC)| >1 and P<0.05 (24, 25). The chromosomal localization of eight types of RNA modification regulator genes was visualized using the R circos package (26).

### Random forest analysis and the screening of feature genes

To anticipate the risk and severity of OA, we constructed a training model adopting both support vector machine (SVM) and RF methods. After comparing the accuracy of the two models, the RF method was then selected to screen candidate RNA modification regulators using the R library “randomForest” with “mtry” and “ntree” setting to 3 and 500, respectively (27). The best “ntree” was selected according to the minimum cross-validation error of 10-fold cross-validation, and the significance between the differentially expressed RNA modification regulators and the best “ntree” were evaluated. We then constructed a nomogram using the ‘rms’ package (28, 29) and performed three kinds of analyses to evaluate the efficacy of our model.

### Hub genes identification

With the aid of box plots, hub gene expression levels in OA and healthy individuals were evaluated. The pROC package in R was used to generate the receiver operating characteristic (ROC) curve and the area under the ROC curve (AUC) was calculated in order to assess the diagnostic performance of each candidate gene. Furthermore, a distinct external dataset was used to validate the hub genes’ expression levels and diagnostic utility (GSE55457).

*In situ* synovial tissues from 3 patients with meniscal injuries and OA were collected through arthroscopy in Huashan hospital. The study followed the guidelines of the 1975 Declaration of Helsinki and was approved by the ethics committee of Huashan Hospital (KY2020-060). Using Trizol, total RNA was extracted from synovial tissue samples (Thermo, California, USA). Following quality checks, PrimeScriptTM RT Master Mix was used to reverse-transcribe total RNA to complementary DNA (cDNA) (TaKaRa, Tokyo, Japan). SYBR Green Master Mix (Thermo, California, USA) and cDNA were used in accordance with the manufacturer’s instructions to perform qRT-PCR for pertinent genes. **Table S2** displays the target genes’ primer sequences. Genes were adjusted to GAPDH’s value. Using the 2^–ΔΔCT^ approach, the relative expression of mRNA was determined. Each experiment was carried out three times as technical replicates.

### Identification of RNA modification clusters

Unsupervised consensus clustering was conducted applying the ‘ConsensusClusterPlus’ package to identify RNA modification (RM) clusters based on 44 RNA modification regulators (30). The principal component analysis (PCA) was conducted to verify the results (31).

### Single-sample gene-set enrichment analysis

The ssGSEA method was performed to assess the infiltration of 23 immune cells in the two distinct RM clusters (32).

### Functional enrichment analysis of DEGs between RM distinct phenotypes

Gene Ontology (GO) analysis (www.geneontology.org/) and Kyoto Encyclopedia of Genes and Genomes (KEGG) analysis (www.genome.jp/kegg/pathway.html) of DEGs between the two RM clusters were conducted using the ‘clusterProfiler’ and ‘enrichplot’ packages of R (25). The P value < 0.05 was regarded as significantly enriched.

### Generation of RNA modification score

We then generated gene clusters applying consensus clustering based on the DEGs between two RM clusters. DEGs between OA and normal subjects were then selected to construct a scoring system using PCA. Each principal component 1 was added for score calculation.


$$\text{Rmscore}=\sum \text{PC}1_{\text{i}}$$

### Correlation between an RNA modification gene signature and immune filtration and other related biological processes

We analyzed the correlation between gene clusters and RNA modification, Rmscore, immune filtration and senescence process. The infiltration of 23 immune cells in the two RNA modification gene clusters was assessed using the ssGSEA method to further demonstrate the connection between the RNA modification gene signature and immune cells. The gene expression of some OA-related inflammatory cytokines and senescence cytokines in the two gene clusters and the two RM clusters was then identified and visualized.

### Statistical analysis

All statistical analyses in our study were conducted with R software, version 4.1.1. The Wilcoxon test was performed for groups comparisons, and an adjusted P < 0.05 was defined as a significant difference. For all figures: * represents p < 0.05, ** represents p < 0.01, and *** represents p < 0.001.

## Results

### The relationships between eight-type RNA modification regulators and OA

**Figure 1:**
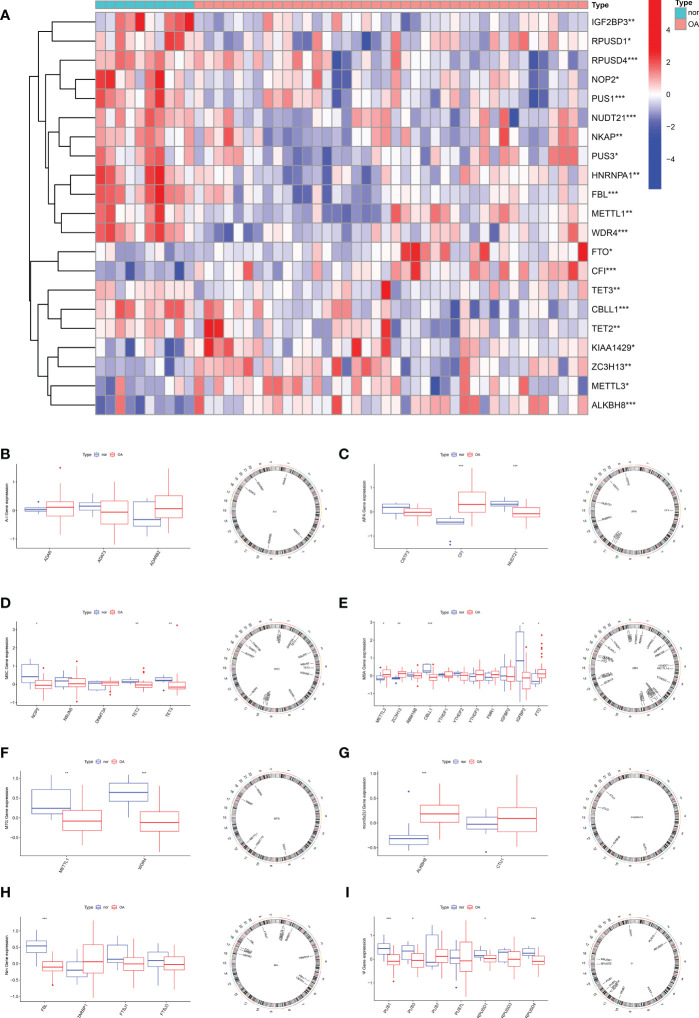
Expression characteristics and gene localization of RNA modification regulators. **(A)** Heat map showing the expression characteristics of RNA modification regulators in OA tissues and normal tissues. Red shows high expression levels while blue shows low expression levels; **(B–I)** box plot showing differences in the expression of A-I, APA, m5C, m6A,m7G, mcm5s2U, Nm and Ψ modification regulators in OA and normal tissues, and the position of their genes on the chromosome, respectively. (for all figures: * represents p <0.05, ** represents p <0.01 and *** represents p <0.001). OA, osteoarthritis, A-I, adenosine-to-inosine; APA, alternative polyadenylation.

To elucidate the RNA modification changes associated with OA, differential expression analysis was performed using data in the GSE51588 dataset. Then, we extracted a gross of 21 RNA modification DEGs in OA patients (n=30) and normal subjects (n=10). **Figure 1A** demonstrated the distribution of DEGs between OA patients and healthy subjects. Analysis showed that *METTL3* (P<0.05), *FTP* (P<0.05), *ZC3H13* (P<0.01), *CFI* (P<0.001), *CBLL1* (P<0.001) and *ALKBH8* (P<0.001) expressed higher in OA. In contrast, *NOP2* (P<0.05), *IGFBP3* (P<0.05), *PUS3* (P<0.05), *RPUSD3* (P<0.05), *TETE2* (P<0.01), *TET3* (P<0.01), *METTL1* (P<0.01), *RPUSD4* (P<0.001), *PUS1* (P<0.001), *NUDT21* (P<0.001), *FBL* (P<0.001) and *WDR4* (P<0.001) were expressed at low levels in OA. Boxplots were used to display the differences in gene expression for each type of RNA modification between groups; circos plots were also used to show the position of genes on the chromosomes (**Figures 1B–I**).

### The construction of predictive models for OA using the SVM and RF methods

**Figure 2:**
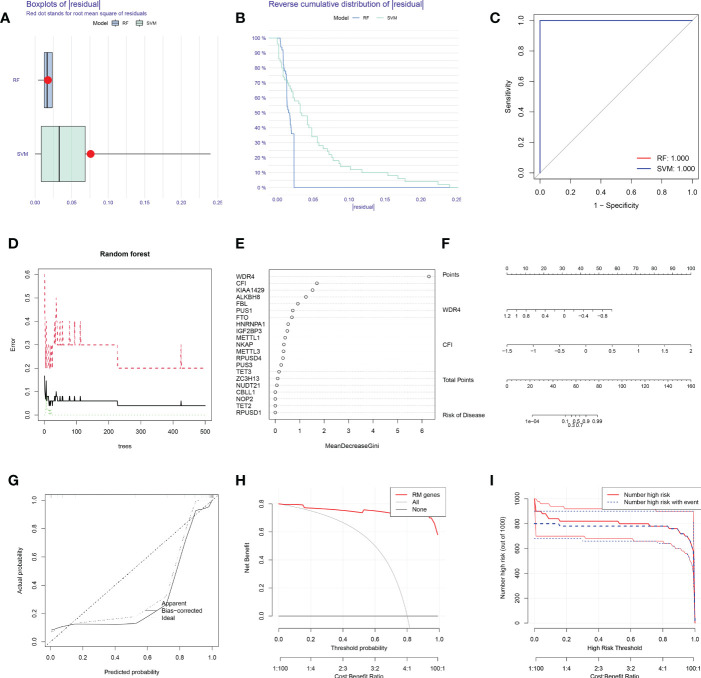
SVM and RF methods were used to construct OA predictive models. **(A, B)** Boxplot of the residual distribution **(A)** and reverse cumulative distribution of residuals **(B)** as a function of the values of observed sensitivity between RF and SVM. **(C)** ROC curves showing predictions for the SVM and RF models. **(D)** RF prediction error curves based on 10-fold cross-validation. **(E)** The importance of the 21 RNA modification regulators based on the RF model. **(F)** Nomogram of the predictive model based on two RNA modification regulators. **(G)** Calibration curves showing that the nomogram model may be an ideal predictive model for OA. **(H, I)** DCA **(H)** and clinical impact plots **(I)** were used to determine the clinical utility of the risk prediction nomograms. SVM, support vector machine; RF, random forest; OA, osteoarthritis; ROC, receiver operating characteristic; DCA, decision curve analysis; RM, RNA modification.

Boxplots of residuals (**Figure 2A**), reverse cumulative distribution of residuals (**Figure 2B**), and ROC curve analysis (**Figure 2C**) revealed that RF exhibited significantly high predictive capability. According to the minimum cross-validation error in 10-fold cross-validation, the best ‘ntree’ was selected (**Figure 2D**). In total, we identified 21 RNA modification regulators and ranked them according to their importance (**Figure 2E**).

To predict the probability of OA, we constructed a nomogram evaluation mode based on 2 RNA modification regulators (*WDR4* and *CFI*) (**Figure 2F**). Calibration curves (**Figure 2G**), decision curve analysis (DCA) (**Figure 2H**) and clinical impact plots (**Figure 2I**) proved the nomogram to be an ideal model for OA.

### Identification of hub gene expression levels and diagnostic value

**Figure 3:**
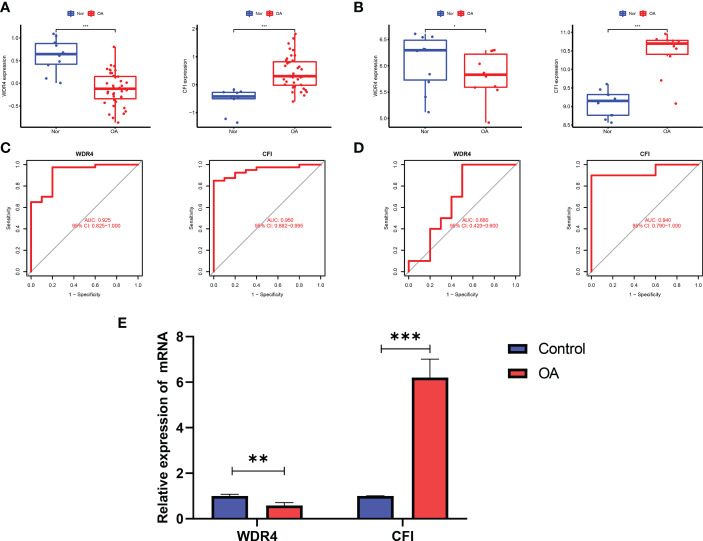
Validation of hub genes in the gene expression level and diagnostic value. **(A)** Validation of hub genes in the GSE51588. *CFI* were significantly more highly expressed in OA compared with healthy controls, while *WDR4* was significantly lower expressed in OA tissues compared with healthy controls. **(B)** Validation of hub genes in the GSE55457 and the results were the same as the results of the GSE51588. **(C)** Validation of hub genes in the GSE51588. ROC curves and AUC statistics are used to evaluate the capacity to discriminate OA from healthy controls with excellent sensitivity and specificity. **(D)** Validation of hub genes in the GSE55457 and the results were similar to the results obtained from GSE51588. **(E)** The relative mRNA expression of *WDR4* and *CFI* were displayed. For all figures: * represents p <0.05, ** represents p <0.01 and *** represents p <0.001. OA, osteoarthritis; ROC, receiver operating characteristic; AUC, area under the curve.

In contrast to healthy controls, OA tissues had significantly greater levels of *CFI* and lower levels of *WDR4* (**Figure 3A**). The expression levels of these four hub genes were then further verified in the GSE55457 external dataset (**Figure 3B**).

The two hub genes’ AUC values were compared for ROC curve analysis in order to evaluate their sensitivity and specificity for the diagnosis of OA. AUC values > 0.6 for all hub genes indicated their relatively good diagnostic value for OA (**Figure 3C**). The diagnostic value of the four hub genes listed above was further confirmed in the GSE55457 dataset to ensure their generalizability (**Figure 3D**).

In our qRT-PCR analyses, we also found that *CFI* were more highly expressed in OA, while *WDR4* was higher in healthy controls (**Figure 3E**).

### Identification of two distinct RM clusters

**Figure 4:**
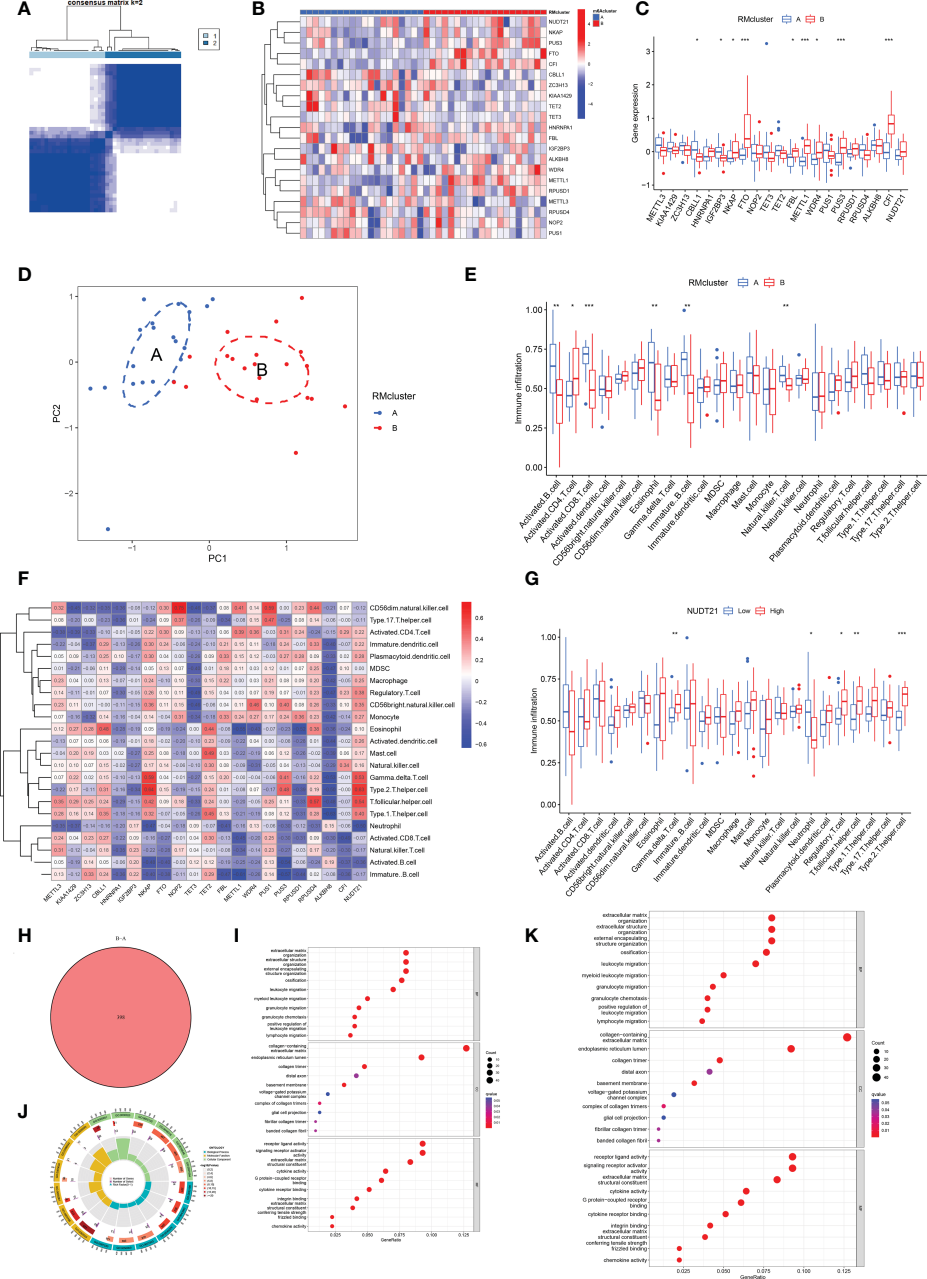
Identification of two distinct RM clusters, immune cell infiltration, and GO and KEGG pathway enrichment analyses. **(A)** Consensus clustering matrix of OA samples for k=2. The OA patients were divided into two clusters: RM clusters A and B. **(B, C)** Boxplot **(B)** and heat map **(C)** showing differential gene expression in the two RM clusters. **(D)** PCA was used to verify the two distinct RM clusters. **(E)** Box plot showing the infiltrating immune cells in the two RM clusters. **(F)** Heat map of the correlation between the expression of the 21 RNA modification regulators and immune cells infiltration by the ssGSEA method. **(G)** WDR4 was negatively correlated with activated B cells (P<0.05), immature B cells (P<0.05) and eosinophils (P<0.01) and positively related to activated CD4^+^ T cells (P<0.05) and CD56 bright natural killer cells (P<0.05). **(H)** Venn diagram showing 398 differential genes between the two RM clusters. **(I)** Bubble diagram showing the top 10 terms of GO categories for BP, MF and CC. **(J)** The circlize diagram showing GO term analysis for BP, MF and CC. The first lap indicates the top 10 GO terms; the number of genes corresponds to the outer lap. The second lap indicates the number of genes in the genome background and P values for enrichment of the differential genes for the specified BP, MF and CC terms. The third lap indicates the number of selected genes for each GO term. The fourth lap indicates the enrichment factor for each GO term. **(K)** Bubble diagram showing KEGG enrichment analysis of differential genes between the two RM clusters. (for all figures, * represents p <0.05, ** represents p <0.01 and *** represents p <0.001) RM, RNA modification; OA, osteoarthritis; PCA, principal component analysis; GO, gene ontology; KEGG, Kyoto Encyclopedia of Gene and Genome; BP, biological process; MF, molecular function; CC, cellular component.

We identified two RM clusters (RM cluster A and RM cluster B) based on 21 RNA modification regulators (**Figure 4A**). **Figure 4B** displayed differentially expressed genes in the two RM clusters. Specifically, the boxplot showed that *CBLL1* (P<0.05) and *IGF2BP3* (P<0.5) expressed higher in RM cluster A, while *NKAP* (P<0.05), *FBL* (P<0.05), *WDR4* (P<0.05), *FTO* (P<0.001), *METTL1*, (P<0.001), *PUS3* (P<0.001) and *CFI* (P<0.001) in RM cluster B (**Figure 4C**). PCA verified the two RM clusters to be reasonable (**Figure 4D**).

### Immune infiltration in RM clusters

More significant infiltration of activated B cells (P<0.01), activated CD8^+^ T cells (P<0,001), eosinophils (P<0.01), immature B cells (p<0.01) and natural killer (NK) T cells (p<0.01) was detected in RM cluster A (**Figure 4E**). Moreover, we analyzed the correlation between gene expression of the 21 RNA modification regulators and immune infiltration (**Figure 4F**). Specifically, NUDT21 was positively related to gamma delta T cells (P<0.01), regulatory T cells (P<0.05), T follicular helper cells (P<0.01) and type 2 T helper cells (P<0.001), and negatively related to neutrophils (P<0.05) (**Figure 4G**).

### Function enrichment analyses of RM clusters

Venn diagram analysis identified 398 DEGs between the two RM clusters (**Figure 4H**). GO annotation and KEGG pathway analyses were performed based on the DEGs between the two RM clusters to perform gene functional enrichment analysis. Biological process (BP) analysis showed that the DEGs were markedly enriched in extracellular matrix organization, extracellular structure organization, external encapsulating, structure organization, ossification, leukocyte migration, myeloid leukocyte migration, granulocyte migration, positive regulation of leukocyte migration and lymphocyte migration. With regards to the molecular function (MF) of GO terms, DEGs were mostly connected with collagen-containing extracellular matrix, endoplasmic reticulum lumen, collagen trimer, distal axon, basement membrane, voltage-gated potassium channel complex, complex of collagen trimers, glial cell projection, fibrillar collagen trimer and banded collagen fibril. With regards to cellular components (CC), the DEGs were significantly related to receptor ligand activity, signaling receptor activator, extracellular matrix structural constituent, cytokine activity, G protein-coupled receptor binding, integrin binding, extracellular matrix, structural constituents, conferring tensile strength frizzled binding and chemokine activity (**Figures 4I, J**). The KEGG results exhibited that the DEGs were primarily connected with cytokine-cytokine receptor interaction, human papillomavirus infection, protein digestion and absorption, rheumatoid arthritis, ECM-receptor interaction, the Wnt signaling pathway, breast cancer, chemokine signaling pathways, the IL-17 signaling pathway, viral protein interaction with cytokine and cytokine receptor and the NF-kappa B signaling pathway (**Figure 4K**).

### Generation of RNA modification gene clusters

**Figure 5:**
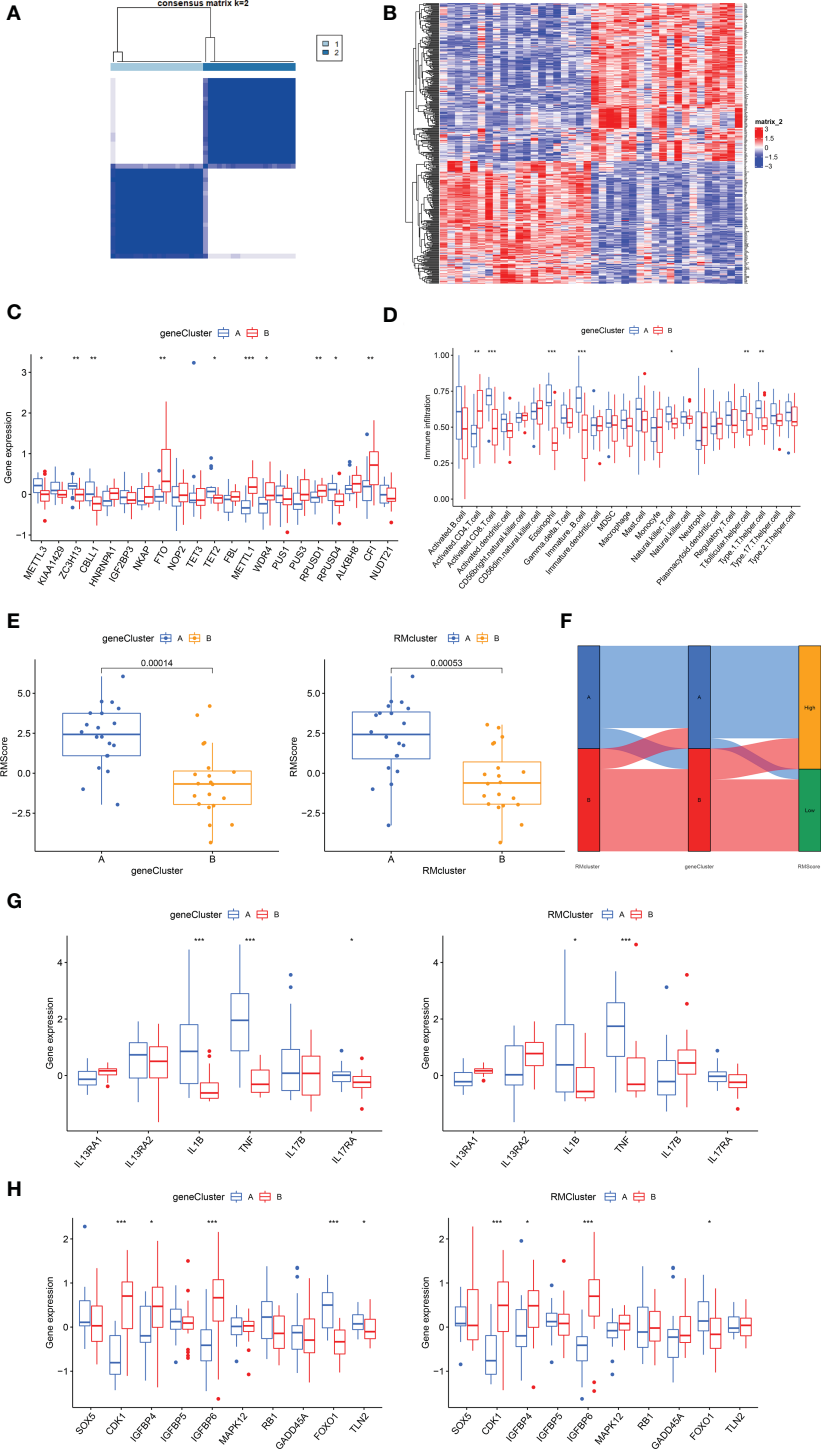
Construction of RNA modification regulator signatures. **(A)** Consensus clustering matrix of overlapping RNA modification regulators phenotype-related genes in OA patients for k=2. The OA patients were classified into different genomic subtypes, termed as gene clusters A and B. **(B)** Heatmap showing the RM cluster differential gene expression for the two gene clusters A and B. **(C)** Boxplot showing the expression of RNA modification regulator differential genes between OA and normal samples. **(D)** Boxplot showing the infiltrating immune cells in the two gene clusters. **(E)** The Rmscore in the two gene clusters and RM clusters. Kruskal-Wallis tests were applied for testing statistical differences. **(F)** Alluvial diagram showing the changes of RM clusters, gene clusters and Rmscore. **(G)** Boxplot showing the immune-related gene expression in the two gene clusters and RM clusters. **(H)** Boxplot showing the senescence-related gene expression in the two gene clusters and RM clusters. (for all figures, * represents p <0.05, ** represents p <0.01, *** represents p <0.001) OA, osteoarthritis; RM, RNA modification; Rmscore, RNA modification score.

To explore the correlation between immune infiltration differences and expression patterns, unsupervised clustering was utilized based on the DEGs between the RM clusters. Clustering results grouped the patients into two gene clusters (gene cluster A and gene cluster B) (**Figure 5A**). A heatmap showing the DEGs in gene clusters A and B (**Figure 5B**). Boxplot showed gene expression differences of 21 RNA modification regulators between the two gene clusters (**Figure 5C**). *METTL3, ZC3H13, CBLL1, TET2* and *ROUSD4* expressed higher in gene cluster A, while *FTO, METTL1, WDR4, RPUSD1* and *CFI* expressed higher in gene cluster B.

### Generation of RNA modification scores

We then constructed the Rmscore using PCA based on DEGs between the two RM clusters to further evaluate individuals’ RNA modification patterns and immune infiltration. Next, we found that Rmscore correlated with RM (P=0.00053) and gene clusters (P=0.00014); RM cluster A and gene cluster A showed higher Rmscores (**Figure 5E**). The changes of individual patients in the GSE51588 dataset were shown in **Figure 5F**.

### Immune infiltration and other functional annotations of RNA modification gene signatures

Similar to RM cluster analysis, more significant infiltration of activated CD8^+^ T cells (P<0,001), eosinophils (P<0.001), immature B cells (p<0.001), NK T cells (p<0.05), T follicular helper cells (P<0.01) and type 1 T helper cells (P<0.01) was detected in gene cluster A (**Figure 5D**). A boxplot was used to show the differences in gene expression of 6 OA-related inflammatory cytokines (*IL13RA1, IL13RA2, IL1B, TNF, IL17B, IL17RA*) and senescence genes (*SOX5, CDK1, IGFBP4, IGFBP5, IGFBP6, MAPK12, RB1, GADD45A, FOXO1 and TLN2*) in the two gene clusters (**Figures 5G, H**). *IL1B, TNF* and *FOXO1*were highly expressed in gene cluster A and RM cluster A; in contrast, *CDK1, IGFBP4* and *IGFBP6* expressed higher in gene cluster B and RM cluster B. In conclusion, RNA modification patterns correlated with an inflamed phenotype.

## Discussion

OA is a progressive and inflammatory disease issue in joint deterioration (33). RNA modification serves as important post-transcriptional regulators participates in the biological processes of eukaryotes and plays a pivotal regulatory role in a variety of diseases (34). However, the mechanisms underlying the relationship between RNA modification and immune cell infiltration in the occurrence and progression of OA have yet to be fully clarified. Our study aimed to investigate the significant role of RNA modification regulators in OA, particularly with respect to RNA modification and immune infiltration, and construct models or scoring systems to quantify RM modification patterns in individuals with high levels of accuracy (35, 36).

A total of 21 RNA modification DEGs between OA (n=30) and normal subjects (n=10) were extracted using a gene expression matrix. Firstly, we detected the levels of 44 RNA modification regulators for total RNAs in OA and normal tissues and found high levels of mcm5s2U in OA tissues. In contrast, the levels of m5C, m7G, Nm and Ψ levels were higher in normal tissues. As for m6A level, *METTL3, ZC3H13* and *FTO* expressed higher in OA, while *CBLL1* and *IGFBP3* expressed higher in normal subjects. In the case of APA level, *CFI* expressed higher in OA while *NUDT21* expressed higher in normal subjects. It was previously reported that m6A participates in OA by controlling the over expression of IL-6 in fibroblast-like synoviocytes (FLS), accelerating the senescence of FLS, inhibiting levels of inflammatory cytokines induced by IL-1βand activating NF-κB signaling in chondrocytes (37, 38).

In the present study, we used SVM and RF methods to screen out genes associated with risk. RF exhibited substantially high predictive accuracy when compared to SVM. We established an RM nomogram to anticipate the occurrence of OA in the light of RNA modification. *CFI* and *WDR4* were identified as hub genes. RT-qPCR yielded consistent results, which confirmed our findings. Different scores were distributed to risk genes such as *WDR4* and *CFI*. The factor scores were summed to obtain the total score. If the gross score was no more than 50, the possibility of occurrence of OA was no more than 0.1; and if the gross score was no less than 70, the possibility of OA was no less than 0.9.

Two genes- *WDR4* and *CFI* were identified as Rmr hub genes in OA. M7G is a highly conserved RNA modification found in tRNA, rRNA, mRNA 5′cap, and internal mRNA regions, and plays a pivotal role in regulating RNA processing, metabolism, and function (39). *WDR4*, a constituent of the human m7G tRNA methyltransferase complex, has been found to cause impaired tRNA m7G modification and be associated with multiple diseases (40–42). Lin et al.’s research showed that *METTL1/WDR4*-mediated m7G tRNA methylome is required for normal mRNA translation and embryonic stem cell self-renewal and differentiation (42). *METTL1/WDR4* was reported to have a strong regulatory effect on cancer (43). An essential post-transcriptional regulation mechanism known as alternative polyadenylation (APA) transforms RNA products based on signals from their 3′-untranslated region (3′-UTR) (44). CFI (cleavage factors I) are made up of CFIm25, CFIm59, and CFIm68, which bind upstream of the conserved UGUA motif to facilitate the cleavage reaction. CFIm25 is also known as NUDT21/nudix hydrolase 21/CPSF5. By looping out the entire pA region and causing the choice of an APA site, CFIm binding can act as a main determinant of pA sites (44). Human haematological, immunological, neurological, and cancerous illnesses all often modify poly(A) site use (45). For example, immunodysregulation polyendocrinopathy enteropathy X-linked (IPEX) syndrome is a primary immunodeficiency and the dysfunction arises from mutations in FOXP3 leading to APA (45). However, there is a significant lack of research relating to *WDR4* and *CFI* in OA; researchers should investigate the possibility of *WDR4* and *CFI* as a novel biomarker for OA in the future.

We classified our OA patients into two Rmr clusters, gene clusters and generated Rmscores for individuals. Most patients in RM cluster A were further divided into gene cluster A and high Rmscore group; while patients in RM cluster B were classified into gene cluster B and low Rmscore group. Our conclusions suggested that RM cluster A significantly correlated with an inflamed phenotype whereas cluster B was strongly correlated with a non-inflamed phenotype. Following enrichment analyses of the two RM clusters demonstrated that RM regulator expression modes significantly correlated with biological processes related to immuno-inflammatory regulation and tissue remodeling. Significantly greater numbers of infiltrating activated B cells (P<0.01), immature B cells (p<0.01), activated CD8^+^ T cells (P<0,001), natural killer (NK) T cells (p<0.01) and eosinophils (P<0.01) were found in RM cluster A. Cellular infiltration in inflamed OA tissue has been reported to be characterized by activated B cells, for example a study detected antibodies against cartilage components, native G1 domain of aggrecan and triosephosphate isomerase (TPI) in OA patients (46); these may be important mechanisms in cartilage degeneration in OA. T cells are involved in the pathogenesis of OA because significant T cell abnormalities have been detected in peripheral blood, synovial fluid, and synovial membrane of OA patients (47). In addition, bioinformatic analysis has demonstrated that eosinophils may participate in OA progression (48); however, the specific function of eosinophils has yet to be fully elucidated (49). The classification of OA patients into inflamed and non-inflamed groups based on RNA modification regulators could be of benefit to early-diagnosis, prognosis, the individual-based treatment of OA while also enhancing our comprehension of the pathogenesis of OA and facilitating the discovery of new targets for immunotherapy.

In addition, some senescence-related genes in OA were compared in the two RM clusters and gene clusters. *CDK1, IGFBP4* and *IGFBP6* expressed higher in gene cluster B and RM cluster B, whereas *FOXO1* was expressed at higher levels in gene cluster A and RM cluster A. *CDK1* is necessary for the normal proliferation of chondrocytes; the deletion of it results in accelerated chondrocyte differentiation (50). The effects of IGFBPs which is independent of IGF includes cell adhesion, growth and apoptosis (51). *FOXO1* is an important regulator in cartilage growth and tissue homeostasis and in aged tissue and OA cartilage its expression is decreased (52). Individuals with an inflammatory phenotype are in a metabolically active state that inhibits cells from progressing into aging and death. Therefore, RM cluster A featured an inflamed and a more senescence signature while RM cluster B was associated with a non-inflamed and a less senescence phenotype.

However, our study still has some limitations that need to be illustrated. First, as the data source was a public database, the sample number of control group and OA group didn’t equivalent, and input mistakes could not be determined. Second, RT-qPCR was used to confirm the distinct expression patterns between OA and healthy samples. However, additional experiments like flow cytometry and single-cell sequencing are still required to elucidate the mechanism in detail.

Our research was the first to systematically investigate the crosstalk values for eight-type RNA modifiers in immune landscape during the progression of OA. *WDR4* and *CFI* were distinguished as novel biomarkers and utilized to construct an OA predictive model. Two different RNA modification modes and their connection with immune infiltration were revealed, and a novel scoring system to quantize RM modification modes in individuals was constructed. Our study emphasized the importance of eight-type RNA modifications in OA and offered a new perspective for future studies of OA.

## Data availability statement

The datasets presented in this study can be found in online repositories. All data used in this work were obtained from the GEO (https://www.ncbi.nlm.nih.gov/geo/). The accession number(s) can be found in the article/**Supplementary Material**.

## Ethics statement

The study followed the guidelines of the 1975 Declaration of Helsinki and was approved by the ethics committee of Huashan Hospital (KY2020-060). The patients/participants provided their written informed consent to participate in this study.

## Author contributions

ZC, WW and YH designed and directed the study. ZC and WW performed data analyses. ZC and WW wrote this manuscript and revised the manuscript. YH supervised and managed the entire study process. All authors approved this manuscript. All authors contributed to the article and approved the submitted version.
