# Supplementary material for: Expression patterns of eight RNA-modified regulators correlating with immune infiltrates during the progression of osteoarthritis
Source: Front Immunol. 2023 Mar 15;14:1019445. doi: 10.3389/fimmu.2023.1019445 (PMC10050518; doi:10.3389/fimmu.2023.1019445)
Supplement: Supplementary file 1 [file Table_1.pdf]

Table S1. 109 RNA regulators of eight types of RNA modifications included in this study.

Gene	Modification	Type
METTL3	M6A	Writer
METTL14	M6A	Writer
METTL16	M6A	Writer
WTAP	M6A	Writer
KIAA1429	M6A	Writer
RBM15	M6A	Writer
RBM15B	M6A	Writer
ZCCHC4	M6A	Writer
ZC3H13	M6A	Writer
METTL5	M6A	Writer
CBLL1	M6A	Writer
YTHDF1	M6A	Reader
YTHDF2	M6A	Reader
YTHDF3	M6A	Reader
YTHDC1	M6A	Reader
YTHDC2	M6A	Reader
HNRNPA1	M6A	Reader
HNRNPA2B1	M6A	Reader
HNRNPC	M6A	Reader
IGF2BP1	M6A	Reader
IGF2BP2	M6A	Reader
IGF2BP3	M6A	Reader
NKAP	M6A	Reader
EIF3A	M6A	Reader
FMR1	M6A	Reader
ELAVL1	M6A	Reader
G3BP1	M6A	Reader
G3BP2	M6A	Reader
PRRC2A	M6A	Reader
RBMX	M6A	Reader
LRPPRC	M6A	Reader
FTO	M6A	Eraser
ALKBH5	M6A	Eraser
NSUN2	M5C	Writer
NSUN3	M5C	Writer
NSUN6	M5C	Writer
NSUN7	M5C	Writer
NOP2	M5C	Writer
NSUN4	M5C	Writer
NSUN5	M5C	Writer

DNMT1	M5C	Writer
TRDMT1	M5C	Writer
DNMT3A	M5C	Writer
DNMT3B	M5C	Writer
ALYREF	M5C	Reader
YBX1	M5C	Reader
TET1	M5C	Eraser
TET3	M5C	Eraser
TET2	M5C	Eraser
TRMT6	M1A	Writer
TRMT61A	M1A	Writer
TRMT61B	M1A	Writer
TRMT10A	M1A	Writer
TRMT10B	M1A	Writer
TRMT10C	M1A	Writer
RRP8	M1A	Writer
ALKBH1	M1A	Eraser
ALKBH3	M1A	Eraser
CMTR1	Nm	Writer
FBL	Nm	Writer
TRMT44	Nm	Writer
TRMT13	Nm	Writer
TARBP1	Nm	Writer
FTSJ1	Nm	Writer
SNORD48	Nm	Writer
HENMT1	Nm	Writer
CMTR2	Nm	Writer
FBLL1	Nm	Writer
FTSJ3	Nm	Writer
MRM1	Nm	Writer
MRM2	Nm	Writer
MRM3	Nm	Writer
TRMT11	Nm	Writer
METTL1	M7G	Writer
WDR4	M7G	Writer
BUD23	M7G	Writer
RNMT	M7G	Writer
TRMT112	M7G	Writer
TGS1	M7G	Writer
PUS1	Ψ	Writer
PUS3	Ψ	Writer
TRUB1	Ψ	Writer
PUS7	Ψ	Writer

PUS10	Ψ	Writer
PUS7L	Ψ	Writer
RPUSD1	Ψ	Writer
RPUSD2	Ψ	Writer
RPUSD3	Ψ	Writer
RPUSD4	Ψ	Writer
DKC1	Ψ	Writer
ADAR	A-to-I	Writer
ADARB1	A-to-I	Writer
ADAT2	A-to-I	Writer
ADAT3	A-to-I	Writer
ADARB2	A-to-I	Writer
ELP1	mcm5s2U	Writer
ELP3	mcm5s2U	Writer
ALKBH8	mcm5s2U	Writer
CTU1	mcm5s2U	Writer
CTU2	mcm5s2U	Writer
CPSF1-4	APA	Writer
CSTF1	APA	Writer
CSTF2	APA	Writer
CSTF3	APA	Writer
PCF11	APA	Writer
CFI	APA	Writer
CLP1	APA	Writer
NUDT21	APA	Writer
PABPN1	APA	Writer
