# Supplementary material for: Expression patterns of eight RNA-modified regulators correlating with immune infiltrates during the progression of osteoarthritis
Source: Front Immunol. 2023 Mar 15;14:1019445. doi: 10.3389/fimmu.2023.1019445 (PMC10050518; doi:10.3389/fimmu.2023.1019445)
Supplement: Supplementary file 2 [file Table_2.docx]

Table S1 Primers employed in this study

| Gene symbol | Forward primer (5'-3') | Reverse primer (5'-3') |
| --- | --- | --- |
| WDR4 | AGCCCTGACTTTCATAGCCTCG | TGAGTTGGCACCACGGAGATA |
| CFI | CCGTATCAGTGCCCAAAGAAT | CCATGCTTCAAGGAAACACTAAAC |
